# Retraction: Cleft Lip and Palate Associated With the Phonetic Function: A Systematic Review

**DOI:** 10.7759/cureus.r230

**Published:** 2026-05-18

**Authors:** Anahí Montesdeoca, Dennys Tenelanda Lopez, Mauro Costales, Alejandro Pallo, Sandra Quisiguina

**Affiliations:** 1 Dentristy, Ministry of Public Health, Ambato, ECU; 2 School of Dentistry, National University of Chimborazo, Riobamba, ECU; 3 School of Nutrition and Dietetics, Higher Polytechnic School of Chimborazo, Riobamba, ECU

This article has been retracted by the Editor-in-Chief due to concerns regarding the accuracy and interpretation of data derived from previously published studies. Post-publication review identified errors in the reporting and interpretation of referenced data. These issues affect the reliability of the analyses and conclusions presented in the article.

